# Investigating the relationship between cancer and orofacial clefts using GWAS significant loci for cancers: A case-control and case-triad study

**DOI:** 10.3389/froh.2022.915361

**Published:** 2022-08-05

**Authors:** Azeez Fashina, Tamara Busch, Mary Young, Olawale Adamson, Waheed Awotoye, Azeez Alade, Chinyere Adeleke, Mohaned Hassan, Abimbola M. Oladayo, Lord J. J. Gowans, Mekonen Eshete, Thirona Naicker, Joy Olotu, Wasiu L. Adeyemo, Azeez Butali

**Affiliations:** ^1^Department of Oral and Maxillofacial Surgery, University of Lagos, Lagos, Nigeria; ^2^Department of Oral Pathology, Radiology and Medicine, College of Dentistry, University of Iowa, Iowa City, IA, United States; ^3^Iowa Institute of Oral Health Research, University of Iowa, Iowa City, IA, United States; ^4^Komfo Anokye Teaching Hospital, Kwame Nkrumah University of Science and Technology, Kumasi, Ghana; ^5^Department of Surgery, School of Medicine, Addis Ababa University, Addis Ababa, Ethiopia; ^6^School of Clinical Medicine, KwaZulu-Natal University, Durban, South Africa; ^7^Department of Anatomy, University of Port Harcourt, Port Harcourt, Nigeria

**Keywords:** cleft, lip, palate, oral cancer, orofacial, genetic, genomic

## Abstract

**Background:**

Several population-based case-control studies have reported concurrent presentation of cancer and congenital malformations. Many associations have been made between oral clefting and cancers, though some of these results are conflicting. Some studies have reported an increased risk of cancer among 1st-degree relatives of cleft cases and vice versa, and also an excess risk of cancers of the breast, lung, and brain among those with oral clefts. This study aimed to determine if the genetic polymorphisms found in some cancers are also associated with orofacial cleft in an African cohort.

**Methods:**

The study was a case-control and case-triad study in which cases were 400 individuals clinically diagnosed with non-syndromic cleft lip and/or palate (CL/P), while controls were 450 individuals without CL/P. Samples were obtained from three African countries while DNA extraction, PCR, and genotyping were carried out at the University of Iowa, US. Eleven SNPs in genes coding for SWI/SNF subunits and 13 GWAS significant SNPs for cancers associated with orofacial cleft were selected. Case-control analysis, transmission disequilibrium test (TDT), and DFAM to combine the parent-offspring trio data and unrelated case/control data in a single analysis were carried out using PLINK.

**Results:**

For the case-control analyses that included all the clefts and for the CLP subtype, none of the SNPs were statistically significant. Statistically increased risk for the following SNPs rs34775372 (*p* = 0.02; OR = 1.54, CI:1.07–2.22), rs55658222 (*p* = 0.009; OR = 2.64, CI:1.28–5.45) and rs72728755 (*p* = 0.02; OR=2.27, CI:1.17–4.45) was observed with the CL only sub-group. None of these were significant after Bonferoni correction. In the TDT analyses, a significantly reduced risk with rs10941679 (*p* = 0.003; OR = 0.43, CI:0.24–0.75) was observed and this was significant after Bonferroni correction. The rs10941679 was also significant (*p* = 0.003) in the DFAM analyses as well even after Bonferroni correction.

**Conclusion:**

The results from this study represent an important starting point for understanding the concurrent presentation of some cancers in orofacial clefts, and cancer risks in cleft patients. The associations observed warrant further investigation in a larger cohort and will set the stage for a more mechanistic approach toward understanding the risk for cancers in families with clefts.

## Introduction

Current knowledge about the causes of orofacial cleft (OFC) points particularly toward genetic risk factors, though environmental factors such as smoking have also been implicated [1]. The study of the pathogenesis of OFC has provided ample opportunities to identify candidate genes for this disorder [2]. Cancers also have a multifactorial etiology, with environmental and genetic factors playing important roles. Recent molecular studies have identified several genetic susceptibility factors for various cancer subtypes [3].

Concurrent presentation of cancer and congenital malformations has been reported in several population-based case-control studies [4]. An established example of this includes the basal cell nevus syndrome, which presents with congenital anomalies and basal cell carcinoma and medulloblastoma. Many associations have been reported between orofacial clefts (OFC) and cancers, though some of these studies are conflicting [5, 6]. Some studies have reported an increased risk of cancer among 1st-degree relatives of cleft cases and vice versa, and also an excess risk of cancers of the breast, lung, and brain among those with oral clefts [7, 8].

Various epidemiological study designs have been applied to identify associations between specific cancer entities and OFC. A study by Bille et al. [9] found a significantly higher prevalence of: breast cancer in females with cleft lip and/or cleft palate; brain cancer in females with cleft palate; and lung cancer in males with cleft lip and palate (CL/P).

Since both cancer and OFC have a multifactorial etiology and are sometimes seen together, they both may share genetic and environmental risk factors. Most published investigations into a common genetic etiology for OFC and cancer have been purely descriptive [10]. Many of them have revealed possible links between OFC and specific cancer subtypes. For example, chromosomal region 8q24.21 contains several cancer-risk single nucleotide polymorphisms (SNPs), and it is also a significant risk locus for non-syndrome cleft lip and palate (NSCL/P**)0.1** [10].

Other studies have found mutations in chromatin remodeling complexes associated with cancers also to be associated with non-cleft craniofacial deformities [11, 12]. Mutations of the *ARID1B* gene (a gene that provides instructions for making a protein that forms one subunit of several different SWItch/Sucrose Non-Fermenting (SWI/SNF) protein complexes) was found by Vals et al. in 2014 to be the cause of Coffin-Siris syndrome. This syndrome is characterized by many variable signs and symptoms, including craniofacial deformities such as a wide nose with a flat nasal bridge, a wide mouth with thick lips, thick eyebrows, and eyelashes. The *ARID1B* protein and other SWI/SNF subunits are thought to act as tumor suppressors. As such, mutations in the *ARID1B* gene have been found to be involved in several types of cancer, including breast cancer, neuroblastoma, and diffuse large B-cell lymphoma [13]. Polymorphisms of another SWI/SNF-related gene, *SMARCA2*, have also been found to be associated with Nicolaides-Baraitser syndrome (which also presents with distinctive facial features) [12] and lung, and head and neck cancers.

The knowledge of a shared genetic predisposition between some cancers and OFC will not only advance our understanding but also give more insight into the functions of culpable genes and genomic regions that may be identified. Most studies that have been carried out on this possible relationship between OFC and cancers have been predominantly on Caucasians. [9, 10]. This present study aimed to determine if the genetic polymorphisms found in some cancers are also associated with orofacial cleft in an African cohort. Specifically, this study focused on GWAS significantly associated polymorphisms for cancers and SNPs associated with chromatin remodeling complexes.

## Methods

### Study design

The study was a case-control and case-triad study in which cases were 400 individuals clinically diagnosed with non-syndromic cleft lip and/or palate (CL/P), while controls were 450 age- and sex-matched individuals without CL/P.

### Study location

This study was part of an ongoing study where over 12,000 saliva samples from Africa were obtained for our cleft projects (AfriCRAN). The samples for cases and controls in this study were obtained at the Cleft clinic of Oral and Maxillofacial surgery at the Lagos University Teaching Hospital, Lagos; Yekatit 12 Hospital Addis Ababa, Ethiopia; and Kwame Nkrumah University of science and technology Ghana. DNA extraction, PCR, and genotyping for cases and controls were done at the Butali Laboratory, University of Iowa, USA. Ethical approval for the study was obtained from the IRB of each of the participating centers - Lagos University Teaching Hospital (ADM/DCST/HREC/VOL.XV/321), Kwame Nkrumah University of Science and Technology (CHRPE/RC/018/13), and Yekatit 12 Hospital Addis Ababa, Ethiopia (003/10/surg).

### Identification of markers

SNPs near genes coding for SWI/SNF subunits associated with susceptibility to head and neck cancers and Coffin-Siris syndrome were selected Eleven SNPs were selected using this method (Table 1). Furthermore, we searched the GWAS catalog (https://www.ebi.ac.uk/gwas/) to identify SNPs (markers) that have been reported to be significantly associated with cancers. We focused on some cancers that were previously reported to be associated with orofacial clefts, e.g., breast, lung, and colorectal cancer (Bille et al.) [9]. Thirteen SNPs were identified using this method (Table 1). Thus, a total of 24 markers were selected and used for this study (Table 1).

**Table 1:** SNPs selected for genotyping.

Chromatin remodeling complex SNPs (Associated with cancers and Coffin-Siris syndrome)	rs387906845 rs387907140 rs796052240 rs796052242 rs876657378 rs281875226 rs875989800 rs387906812 rs387906857 rs281875238 rs281875239
GWAS significant SNPs for cancer subtypes (that are associated with orofacial cleft)	rs11117758 rs13025833 rs6809420 rs34775372 rs10941679 rs10505477 rs6983267 rs7014346 rs11780156 rs55658222 rs72728755 rs3785982 rs138007679

### Sample size and power calculation

Using the power calculation tool developed by [22], the power estimate for this association study of 400 all cleft cases and 450 controls was calculated for one-sided tests under an additive genetic model. For the association of a locus, we chose an α of 0.05 but adjusted this for the number of SNPs tested. In this case, we genotyped 24 SNPs but only 13 were informative and included in the final analyses. This led to an adjusted α of 3.84 × 10^−3^ (Bonferroni-adjusted α of 0.05 for 13 tests). For convenience, we present power estimates for effect sizes of 2.0 and 1.8, which are at the lower end of the range of effect sizes of discovery GWAS. For an α of 3.84 × 10^−3^ and under an additive genetic model, the study has 93.5% power at a minor allele frequency (MAF) of 0.10, and 68% power at an MAF of 0.05, to detect a genotypic relative risk of 2.0. For a genotypic relative risk of 1.8, the study has 79.3% power at a MAF of 0.10.

### Sample processing

Saliva samples were collected from the cases and controls using Oragene tool kits. These kits were labeled and assigned a unique identification number (UNID). Stored saliva samples from the ongoing genetic studies were used. Consent was obtained prior to the collection of saliva samples. The stored saliva samples were anonymized and cannot be traced to any individual participant.

DNA extraction was carried out at the Butali laboratory using the Murray Laboratory protocol (genetics@uiowa.edu). Extracted DNA samples were quantified using Qubit (http://www.invitrogen.com/site/us/en/home/brands/Product-Brand/Qubit.html; Thermo Fisher Scientific, Grand Island, NY). Stocks and working aliquots were made for downstream analyses. For quality control (QC) analysis, we confirmed the reported sex using the Taqman XY genotyping.

### Pre-amplification of DNA and assay of amplified products

We created the 0.2X PreAmp cocktail by combining 40X assay with the low-TE buffer. The 40X assay contained 1.5l for each of the 24 assay markers. The 0.2X PreAmp cocktail was combined with the PreAmp Master Mix (Qiagen product) to make the sample Pre-Mix. This was then added to 2 ng/ul of DNA before running the amplification program. Positive controls were included in the wells before the pre-amplification. The negative controls were not amplified. These controls help to test the quality of the results of calls. Each well contains 4l of the sample Pre-Mix and 1.3 lul DNA. The DNA samples and positive controls were then amplified. Details of the PCR reaction (used to amplify the DNA) conditions (denaturing, annealing, and extending temperatures) are available from Butali laboratories upon request. Each well is then diluted by adding 2 ul low-TE buffer.

The assay plate and samples were prepared. Details of these preparations are available from Butali laboratories upon request. These samples were then sent to the Iowa Institute of Human Genetics (IIHG) for the Fluidigm run and generation of the data. The protocol for this step is available from Butali laboratories upon request.

The data obtained from the Fluidigm run were analyzed using the Fluidigm SNP Genotyping Analysis software. This software produces an SDS plot based on the genotype of the SNPs (markers) at the loci in each sample (Supplementary Figure 1). The genotypes at different loci were called and were included in the association analyses.

### Data analysis

All the samples were divided into four categories: “All clefts”, “Cleft lip alone (CL)”, “Cleft palate alone (CP),” and “Cleft lip and palate (CLP)” in order to identify the risk of cancers in each of the cleft phenotypes.

We conducted three association tests (case-control, transmission disequilibrium test (TDT), and DFAM) to identify a significant association between cancer GWAS significant SNPs and clefts using PLINK (www.cog-genomics.org/plink2). Case-control analyses have increased power to detect significant statistical association and shows the difference in the MAF between cases and controls. However, case-control analyses are prone to population stratification. For this analysis, only the affected cases and match controls were included. The transmission disequilibrium test (TDT) is used to determine over-transmission of the minor allele to detect an association of the minor allele with the cleft types and it is not prone to population stratification. This involves only the affected cases and both parents. Since we have dyads and triads in the cohort, we then used DFAM to combine both (dyads and triads) in the association analyses as well as combine related and unrelated samples.

## Results

A total of 24 single nucleotide polymorphisms were genotyped. Genotypes from 11 SNPs from chromatin remodeling complexes were not informative since they all genotyped as homozygotes for either of the alleles in our cohort. The genotype data for the 13 GWAS significant SNPs were informative and used for the final analyses (Tables 2–**4**).

**Table 2A:** All clefts –control analyses of GWAS significance SNPs for cancer (all clefts, *N* = 424 and controls, *N* = 449).

**CHR**	**SNP**	**A1**	**A2**	**MAF_A**	**MAF_U**	**OR**	**SE**	**L95**	**U95**	**P**
1	rs11117758	A	G	0.1459	0.1512	0.9092	0.2054	0.6079	1.36	0.64
2	rs13025833	A	G	0.3529	0.3669	0.9338	0.1099	0.7528	1.158	0.53
3	rs6809420	T	G	0.1945	0.1969	1.053	0.1698	0.7551	1.469	0.76
4	rs34775372	T	C	0.2763	0.2298	1.26	0.146	0.9463	1.677	0.11
5	rs10941679	G	A	0.1639	0.1962	0.795	0.1758	0.5632	1.122	0.19
8	rs10505477	G	A	0.1348	0.12	0.943	0.2403	0.5887	1.51	0.81
8	rs6983267	T	G	0.05238	0.04362	1.041	0.4098	0.4664	2.325	0.92
8	rs7014346	A	G	0.3866	0.4049	0.9472	0.1042	0.7722	1.162	0.60
8	rs55658222	A	G	0.09547	0.06264	1.74	0.3405	0.8928	3.392	0.10
8	rs72728755	A	T	0.09308	0.06585	1.501	0.3084	0.82	2.747	0.19

*CHR, chromosome; SNP, variant identifier; A1, Allele 1 (usually minor); A, Allele 2 (usually major); MAF_A, Allele freq in cases; MAF_U, Allele freq in controls; SE, Standard error*.

**Table 2B:** CLP -control analyses of GWAS significance SNPs for cancer (CLP, *N* = 165 and controls, *N* = 449).

**CHR**	**SNP**	**A1**	**OR**	**SE**	**L95**	**U95**	***P***
1	rs11117758	A	1.006	0.2661	0.5971	1.695	0.98
2	rs13025833	A	0.8269	0.1539	0.6116	1.118	0.22
3	rs6809420	T	0.8466	0.2588	0.5098	1.406	0.52
4	rs34775372	T	1.149	0.1992	0.7773	1.697	0.49
5	rs10941679	G	0.6806	0.2773	0.3953	1.172	0.16
8	rs10505477	G	0.9542	0.3335	0.4963	1.834	0.89
8	rs6983267	T	0.9857	0.5794	0.3166	3.069	0.98
8	rs7014346	A	0.9567	0.1413	0.7253	1.262	0.75
8	rs55658222	A	1.411	0.4592	0.5735	3.47	0.45
8	rs72728755	A	1.216	0.4359	0.5173	2.856	0.65

**Table 2C:** CL -control analyses of GWAS significance SNPs for cancer (CL, *N* = 128 and controls, *N* = 449).

**CHR**	**SNP**	**A1**	**OR**	**SE**	**L95**	**U95**	***P***
1	rs11117758	1	1.19	0.27	0.70	2.01	0.52
2	rs13025833	1	1.14	0.15	0.85	1.53	0.39
3	rs6809420	2	1.17	0.23	0.74	1.84	0.50
4	rs34775372	2	1.54	0.18	1.07	2.22	0.02
5	rs10941679	2	0.80	0.28	0.46	1.38	0.41
8	rs10505477	2	1.21	0.30	0.67	2.18	0.53
8	rs6983267	2	1.54	0.46	0.63	3.80	0.34
8	rs7014346	1	0.83	0.15	0.61	1.12	0.22
8	rs55658222	1	2.64	0.37	1.28	5.45	0.009
8	rs72728755	2	2.27	0.34	1.17	4.45	0.02

**Table 2D:** CP -control analyses of GWAS significance SNPs for cancer (CP, *N* = 82 and controls, *N* = 449).

**CHR**	**SNP**	**A1**	**OR**	**SE**	**L95**	**U95**	***P***
1	rs11117758	1	0.57	0.5217	0.21	1.59	0.28
2	rs13025833	1	0.92	0.2031	0.62	1.37	0.69
3	rs6809420	2	1.35	0.2473	0.83	2.19	0.23
4	rs34775372	2	1.26	0.2622	0.76	2.11	0.37
5	rs10941679	2	0.97	0.2813	0.56	1.68	0.90
8	rs10505477	2	0.73	0.5287	0.26	2.07	0.56
8	rs7014346	1	1.09	0.1999	0.73	1.61	0.68

### Case-control analyses

For the analyses that included all the clefts and for CLP subtype, none of the SNPs was statistically significant (Table 2). However, we observed statistically increased risk for the following SNPs rs34775372 (54% increased risk for cancer in CL cases compared to controls), rs55658222 (two-fold increased risk for cancer in CL cases compared to controls), and rs72728755 (two-fold increased risk for cancer in cases compared to controls). None of these were significant after Bonferroni correction. However, the trend toward significance is worthy of note and where the sample size is increased might be clinically relevant.

### TDT analyses

For all clefts, we observed a significantly reduced risk for cancer with rs10941679 (75% reduced risk of cancer in families with all clefts compared with controls) suggesting that this locus might be protective. This was significant after Bonferroni correction. We observed a 7-fold risk for cancer with rs72728755 in families with CLP and CL compared to controls. We also observed a nominally significant 67% reduced risk for cancer in families with CP compared with controls for SNP rs11117758 after Bonferroni correction (Table 3).

**Table 3A:** Transmission disequilibrium test for all clefts (*N* = 156 triads).

**CHR**	**SNP**	**A1**	**T**	**U**	**OR**	**L95**	**U95**	**CHISQ**	***P***
1	rs11117758	1	5	12	0.42	0.15	1.18	2.882	0.09
2	rs13025833	1	23	14	1.64	0.85	3.19	2.189	0.14
3	rs6809420	2	10	14	0.71	0.32	1.61	0.6667	0.41
4	rs34775372	2	14	18	0.78	0.39	1.56	0.5	0.48
5	rs10941679	2	5	20	0.25	0.09	0.67	9	0.003
8	rs10505477	2	9	3	3	0.81	11.08	3	0.08
8	rs6983267	2	4	2	2	0.37	10.92	0.6667	0.41
8	rs7014346	1	23	26	0.88	0.50	1.55	0.1837	0.67
8	rs55658222	1	5	3	1.67	0.40	6.97	0.5	0.48
8	rs72728755	2	4	4	1	0.25	4.0	0	1

**Table 3B:** Transmission disequilibrium test for CLP (*N* = 156 triads).

**CHR**	**SNP**	**A1**	**T**	**U**	**OR**	**L95**	**U95**	**CHISQ**	***P***
1	rs11117758	1	11	8	1.38	0.55	3.42	0.47	0.49
2	rs13025833	1	17	12	1.42	0.68	2.97	0.86	0.35
3	rs6809420	2	7	8	0.88	0.32	2.41	0.07	0.80
4	rs34775372	2	20	10	2	0.94	4.27	3.33	0.07
5	rs10941679	2	7	12	0.58	0.23	1.48	1.32	0.25
8	rs10505477	2	5	5	1	0.29	3.45	0	1
8	rs7014346	1	15	22	0.68	0.35	1.31	1.32	0.25
8	rs55658222	1	6	1	6	0.72	49.84	3.57	0.06
8	rs72728755	2	7	1	7	0.86	56.89	4.5	0.03

**Table 3C:** Transmission disequilibrium test for CL (*N* = 123 triads).

**CHR**	**SNP**	**A1**	**T**	**U**	**OR**	**L95**	**U95**	**CHISQ**	***P***
1	rs11117758	1	11	8	1.38	0.55	3.42	0.47	0.49
2	rs13025833	1	17	12	1.42	0.68	2.97	0.86	0.35
3	rs6809420	2	7	8	0.88	0.32	2.41	0.07	0.80
4	rs34775372	2	20	10	2	0.94	4.27	3.33	0.07
5	rs10941679	2	7	12	0.58	0.23	1.48	1.32	0.25
8	rs10505477	2	5	5	1	0.29	3.45	0	1
8	rs7014346	1	15	22	0.68	0.35	1.31	1.32	0.25
8	rs55658222	1	6	1	6	0.72	49.84	3.57	0.06
8	rs72728755	2	7	1	7	0.86	56.89	4.5	0.03

**Table 3D:** Transmission disequilibrium test for CP (*N* = 79 triads).

**CHR**	**SNP**	**A1**	**T**	**U**	**OR**	**L95**	**U95**	**CHISQ**	***P***
1	rs11117758	1	4	12	0.33	0.11	1.03	4	0.05
2	rs13025833	1	10	9	1.11	0.45	2.73	0.05	0.82
3	rs6809420	2	8	3	2.67	0.71	10.05	2.27	0.13
4	rs34775372	2	11	4	2.75	0.88	8.64	3.27	0.07
5	rs10941679	2	5	8	0.63	0.20	1.91	0.69	0.41
8	rs10505477	2	5	5	1	0.29	3.45	0	1
8	rs6983267	2	1	2	0.5	0.05	5.51	0.33	0.56
8	rs7014346	1	14	11	1.27	0.58	2.80	0.36	0.55
8	rs55658222	1	1	1	1	0.06	15.99	0	1
8	rs72728755	2	1	1	1	0.06	15.99	0	1

### DFAM analyses

For all clefts, we observed a significant association with rs10941679 (*p* = 0.003). This was still significant after Bonferroni correction. For the CLP sub-group, we observed a significant association with rs10941679 (*p* = 0.01) and for the CL subgroup, we observed a significant association with rs72728755 (*p* = 0.03). Finally, we observed a nominal significant association for rs11117758 with CP. None of these SNPs were significant after Bonferroni correction (Table 4).

**Table 4A:** DFAM of all clefts (cases, *N* = 428 and controls, *N* = 1444).

**CHR**	**SNP**	**A1**	**A2**	**OBS**	**EXP**	**CHISQ**	***P***
1	rs11117758	1	2	30	40.14	4.90	0.03
2	rs13025833	1	2	95	86.26	1.97	0.16
3	rs6809420	2	1	50	48.15	0.13	0.72
4	rs34775372	2	1	77	71.3	0.98	0.32
5	rs10941679	2	1	33	47.94	8.83	0.003
8	rs10505477	2	1	28	25.21	0.57	0.45
8	rs6983267	2	1	8	7.147	0.21	0.65
8	rs7014346	1	2	113	114.3	0.03	0.85
8	rs11780156	2	1	0	0.5	1	0.32
8	rs55658222	1	2	20	13.35	5.97	0.01
8	rs72728755	2	1	19	13.75	3.64	0.06
21	rs138007679	2	1	0	0.6691	0.75	0.39

**Table 4B:** DFAM analyses of CLP (cases, *N* = 167 and controls, *N* = 971).

**CHR**	**SNP**	**A1**	**A2**	**OBS**	**EXP**	**CHISQ**	***P***
1	rs11117758	1	2	6	9.5	2.88	0.09
2	rs13025833	1	2	30	26.5	1.26	0.26
3	rs6809420	2	1	13	14.5	0.31	0.58
4	rs34775372	2	1	21	22	0.12	0.73
5	rs10941679	2	1	10	16.5	6.26	0.01
8	rs10505477	2	1	10	7	3	0.08
8	rs6983267	2	1	5	4	0.67	0.41
8	rs7014346	1	2	32	33.5	0.17	0.68
8	rs55658222	1	2	5	4.5	0.11	0.74
8	rs72728755	2	1	4	4.5	0.11	0.74

**Table 4C:** DFAM analyses of CL (cases, *N* = 130 and controls, *N* = 891).

**CHR**	**SNP**	**A1**	**A2**	**OBS**	**EXP**	**CHISQ**	***P***
1	rs11117758	1	2	14	12.5	0.4737	0.49
2	rs13025833	1	2	23	20	1.2	0.27
3	rs6809420	2	1	11	11	0	1
4	rs34775372	2	1	24	19.5	2.613	0.11
5	rs10941679	2	1	8	12	2.909	0.09
8	rs10505477	2	1	6	6	0	1
8	rs6983267	2	1	1	0.5	1	0.32
8	rs7014346	1	2	25	28	0.9474	0.33
8	rs11780156	2	1	0	0.5	1	0.32
8	rs55658222	1	2	7	4.5	3.571	0.06
8	rs72728755	2	1	8	5	4.5	0.03

**Table 4D:** DFAM analyses of CP (cases, *N* = 82 and controls, *N* = 787).

**CHR**	**SNP**	**A1**	**A2**	**OBS**	**EXP**	**CHISQ**	***P***
1	rs11117758	1	2	4	8	4	0.05
2	rs13025833	1	2	12	11.5	0.05	0.82
3	rs6809420	2	1	8	5.5	2.27	0.13
4	rs34775372	2	1	14	10.5	3.27	0.07
5	rs10941679	2	1	5	6.5	0.69	0.41
8	rs10505477	2	1	5	5	0	1
8	rs6983267	2	1	1	1.5	0.33	0.56
8	rs7014346	1	2	20	18.5	0.36	0.55
8	rs55658222	1	2	1	1	0	1
8	rs72728755	2	1	1	1	0	1

## Discussion

Recent studies have demonstrated a relationship between orofacial cleft and cancers, and several genetic alterations have been found to be associated with NSCL/P and a few cancer subtypes. Our study explored the relationship between orofacial clefts and cancers by genotyping for GWAS significant SNPs associated with cancers in an African cohort of NSCL/P patients and their parents.

Five SNPs (rs34775372, rs72728755, rs55658222, rs10941679, and rs11117758) showed significant association with orofacial clefts in one or two of the different analytical approaches. In both the case-control and TDT analyses, we observed an increased risk for CL and CLP for rs72728755. A nominal significant association was also observed for this SNP in the DFAM analyses. These results suggest that the risk for cancer might be higher in individuals with both cleft types when they carry the risk alleles at this locus. The fact that this SNP was identified using three independent approaches provides evidence of the reproducibility of the outcome. The rs72728755 SNP was previously reported to be significantly (*p* = 2.70 × 10–22, OR=2) associated with orofacial clefts in a study by Yang et al. [14].

While the roles of rs55658222 (8q24.21) and rs72728755 (8q24.21) in orofacial cleft are well documented in the literature, less is known of their functions in cancer. These SNPs have not been directly associated with any cancer subtype but have been found to be in linkage disequilibrium with another SNP rs72728744 (8q24.21). The rs72728744 SNP is associated with lung adenocarcinoma [15]. The rs72728744, rs55658222, and rs72728755 are all located on chromosome 8q24, which several studies have shown to be associated with multiple cancer types [16, 17]. Recent studies demonstrate that the 8q24 loci are within potential enhancers. The enhancers physically interact with the MYC gene, which has a longstanding history in cancer biology [17].

In this study, rs34775372 (4p16.2) (which is previously known to be associated with colorectal and breast cancer) [18] was found to be associated with an increased risk for cancer with CL in the case-control analyses. This is the first time this association is being recorded to the best of our knowledge. The rs34775372 is an intergenic variant located at the cytogenetic region 4p16.2 and mapped to be associated with LINC01396 and STX18-AS1 [18]. These two genes have been reported to play a role in cleft lip and palate (CLP) through the association of an intergenic variant, rs1907989, which is also located in the same cytogenetic region [19]. Therefore, it is possible that rs1907989 and rs34775372 are in linkage disequilibrium and may be carrying the same risk for clefts and cancer. The rs1907989 has not been reported to be associated with any cancer subtype.

The rs10941679 was significant after Bonferroni correction in both the TDT and DFAM analyses with reduced risk for cancer in clefts. This locus on chromosome 5 may be protective against cancer in families with clefts. This SNP was previously reported to increase the risk for breast cancer [20]. Additional association was observed after DFAM analyses for rs11117758 on chromosome 1. This locus was previously reported for increased breast cancer risk [21]. The significant association in families with all clefts (combination of all the cleft sub-groups) suggests an increased risk for breast cancer in these families.

## Limitations

This study has several limitations and thus the findings should be interpreted with caution. First, we have a small sample size for the sub-group analyses. Secondly, we did not include environmental exposure data since smoking and some other environmental exposure variables can increase the risk for cancer and clefts. Though we have questionnaire data on exposure, this is limited by recall bias and thus we did not include the data in the analyses.

## Conclusion

This study represents an essential addition to understanding the concurrent presentation of some cancers in orofacial cleft and cancer risks in cleft patients. The associations observed warrant further investigation in a larger cohort and will set the stage for a more mechanistic approach toward understanding the risk for cancers in families with clefts.

## Data availability statement

The original contributions presented in the study are included in the article/Supplementary materials, further inquiries can be directed to the corresponding author/s.

## Ethics statement

The studies involving human participants were reviewed and approved by Lagos University Teaching Hospital (ADM/DCST/HREC/VOL.XV/321), Kwame Nkrumah University of Science and Technology (CHRPE/RC/018/13), and Yekatit 12 Hospital Addis Ababa, Ethiopia (003/10/surg). Written informed consent to participate in this study was provided by the participants' legal guardian/next of kin.

## Author contributions

AF, WLA, and AB wrote the proposal for the study. OA, AF, JO, and WLA coordinated patient recruitment and sample collection in Nigeria. AF and WLA also contributed to the revised manuscript. LG coordinated patient recruitment and sample collection in Ghana. ME coordinated patient recruitment and sample collection in Ethiopia. AF, TB, MY, AA, WA, CA, MH, and AO took part in the sequencing of the samples. AF, TB, MY, and AB performed the analyses of the results. TN contributed to the revised manuscript. AF wrote the final manuscript. AB supervised the analyses and draft of the final manuscript. All authors contributed to the article and approved the submitted version.

## Funding

This research was supported by the Fogarty International Center of the National Institute of Health under Award number D43TW010134 to AF and National Institutes of Health/National Institute of Dental and Craniofacial Research grants DE28300 to AB.

## Conflict of interest

The authors declare that the research was conducted in the absence of any commercial or financial relationships that could be construed as a potential conflict of interest.

## Publisher's note

All claims expressed in this article are solely those of the authors and do not necessarily represent those of their affiliated organizations, or those of the publisher, the editors and the reviewers. Any product that may be evaluated in this article, or claim that may be made by its manufacturer, is not guaranteed or endorsed by the publisher.

## Author disclaimer

The content is solely the authors' responsibility and does not necessarily represent the official views of the National Institutes of Health.
