# Supplementary figures and images for: Investigating the relationship between cancer and orofacial clefts using GWAS significant loci for cancers: A case-control and case-triad study
Source: Front Oral Health. 2022 Aug 5;3:915361. doi: 10.3389/froh.2022.915361 (PMC9388935; doi:10.3389/froh.2022.915361)

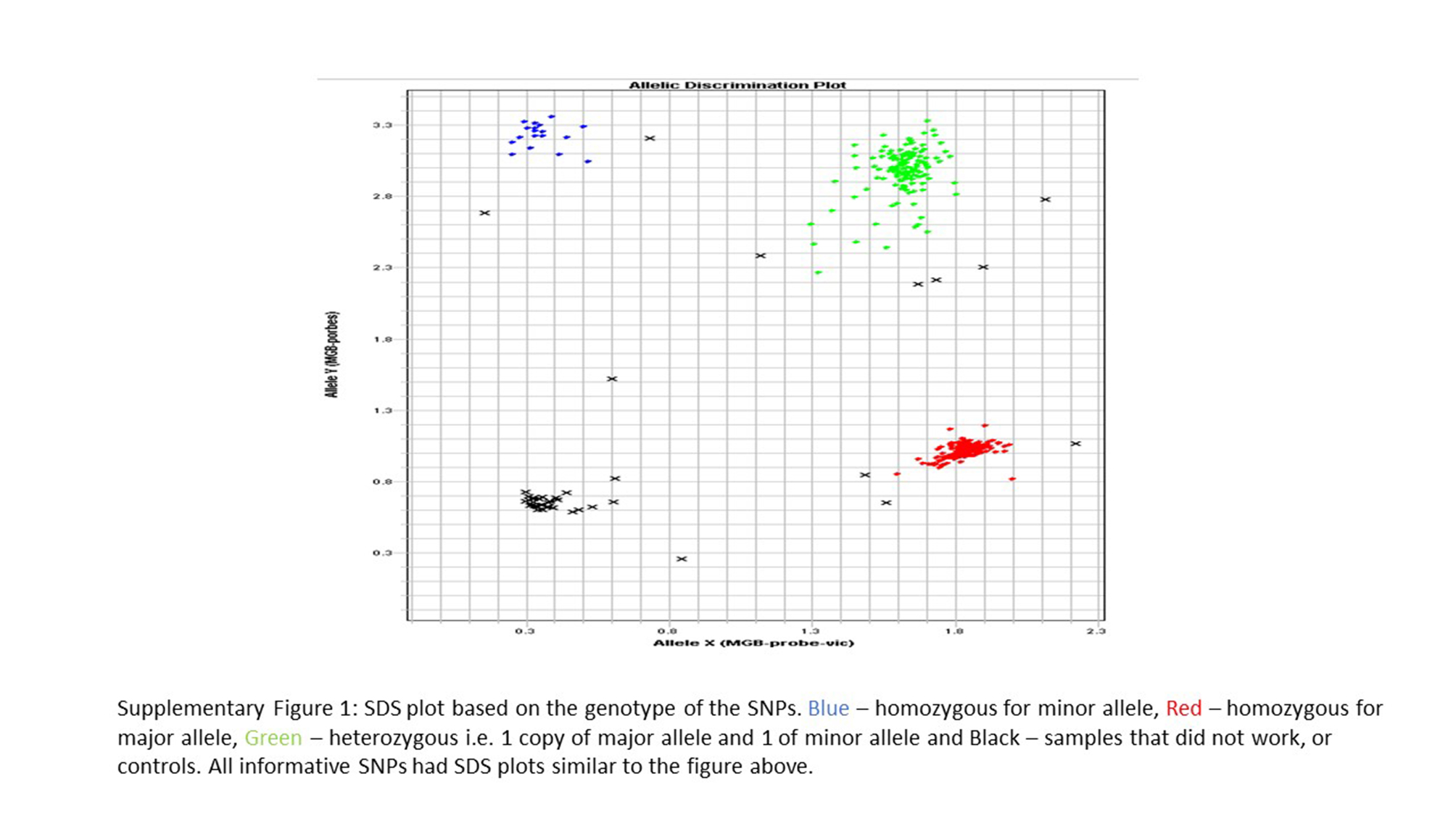

Supplement: Supplementary file 1 [file Image_1.JPEG]
